# Radiogenomics of neuroblastomas: Relationships between imaging phenotypes, tumor genomic profile and survival

**DOI:** 10.1371/journal.pone.0185190

**Published:** 2017-09-25

**Authors:** Hervé J. Brisse, Thomas Blanc, Gudrun Schleiermacher, Véronique Mosseri, Pascale Philippe-Chomette, Isabelle Janoueix-Lerosey, Gaelle Pierron, Eve Lapouble, Michel Peuchmaur, Paul Fréneaux, Louise Galmiche, Nathalie Algret, Matthieu Peycelon, Jean Michon, Olivier Delattre, Sabine Sarnacki

**Affiliations:** 1 Imaging Department, Institut Curie, Paris, France; 2 Paris Sciences et Lettres Research University, Paris, France; 3 Department of Pediatric Surgery, Necker-Enfants-Malades University Hospital, Assistance Publique-Hôpitaux de Paris, Paris, France; 4 Paris-Descartes Sorbonne-Paris-Cité University, Paris, France; 5 Department of Pediatric Oncology, Institut Curie, Paris, France; 6 Unité de Génétique Somatique, Institut Curie, Paris, France; 7 Department of Biostatistics, Institut Curie, Paris, France; 8 Department of Pediatric Surgery and Urology, Robert Debré University Hospital, Assistance Publique-Hôpitaux de Paris, Paris, France; 9 Paris-Diderot Sorbonne-Paris-Cité University, Paris, France; 10 Institut Curie, INSERM, U830, Equipe Labellisée Ligue Contre le Cancer, Paris, France; 11 Department of Pathology, Robert Debré University Hospital, Assistance Publique-Hôpitaux de Paris, Paris, France; 12 Department of Biopathology, Institut Curie, Paris, France; 13 Department of Pathology, Necker-Enfants-Malades University Hospital, Assistance Publique-Hôpitaux de Paris, Paris, France; Universitat Zurich, SWITZERLAND

## Abstract

**Purpose:**

This study investigated relationships between neuroblastomas (NBs) imaging phenotypes, tumor genomic profile and patient outcome.

**Patients and methods:**

This IRB-approved retrospective observational study included 133 NB patients (73 M, 60 F; median age 15 months, range 0–151) treated in a single institution between 1998 and 2012. A consensus review of imaging (CT-scan, MRI) categorized tumors according to both the primarily involved compartment (i.e., neck, chest, abdomen or pelvis) and the sympathetic anatomical structure the tumors rose from (i.e., cervical, paravertebral or periarterial chains, or adrenal gland). Tumor shape, volume and image-defined surgical risk factors (IDRFs) at diagnosis were recorded. Genomic profiles were assessed using array-based comparative genomic hybridization and divided into three groups: “numerical-only chromosome alterations” (NCA), “segmental chromosome alterations” (SCA) and “*MYCN* amplification” (MNA). Statistical analyses included Kruskal–Wallis, Chi^2^ and Fisher’s exact tests and the Kaplan-Meier method with log-rank tests and Cox model for univariate and multivariate survival analyses.

**Results:**

A significant association between the sympathetic structure origin of tumors and genomic profiles was demonstrated. NBs arising from cervical sympathetic chains were all NCA. Paravertebral NBs were NCA or SCA in 75% and 25%, respectively and none were MNA. Periarterial NBs were NCA, SCA or MNA in 33%, 56% and 11%, respectively. Adrenal NBs were NCA, SCA or MNA in 16%, 36% and 48%, respectively. Among MNA NBs, 92% originated from the adrenal gland. The sympathetic anatomical classification was significantly better correlated to overall survival than the compartmental classification (P < .0003). The tumor volume of MNA NBs was significantly higher than NCA or SCA NBs (P < .0001). Patients with initial volume less than 160 mL had significantly better overall survival (P < .009). A “single mass” pattern was significantly more frequent in NCA NBs (P = .0003). The number of IDRFs was significantly higher in MNA NBs (P < .0001).

**Conclusion:**

Imaging phenotypes of neuroblastomas, including tumor origin along the sympathetic system, correlate with tumor genomic profile and patient outcome.

## Introduction

Neuroblastomas (NBs) are the most common extracranial solid tumors in children. NBs derive from the sympathetic nervous system originating from neural crest cells. Therefore, these tumors may theoretically arise from any migratory pathway [1, 2]. NBs mostly arise from the abdomen (adrenal gland 48%, extra-adrenal retroperitoneum 25%), less frequently from the chest (16%) and rarely from the pelvis (3%) or the neck (3%)[3]. NBs are associated with remarkable biological heterogeneity and outcome. Some tumors undergo spontaneous regression, some are cured by surgery alone or after chemo-reduction, while other exhibit extremely aggressive behavior.

Among prognostic factors previously identified, many are associated with each other and define pretreatment risk groups[3]. Major prognostic factors are: the age at diagnosis (better prognosis if younger than 18 months), the tumor stage (according to the International Neuroblastoma Staging System (INSS)[4] or the International Neuroblastoma Risk Grouping Staging System (INRGSS)[5]), the pathology based on the International Neuroblastoma Pathology Classification (INPC)[6–8], various biological factors [3], and somatic genetic abnormalities, especially the amplification of the *MYCN* oncogene, which occurs in 20 to 25% of NBs[9]. Whole-genome DNA copy number analysis with array-based comparative genomic hybridization (aCGH) provided further critical prognostic information, especially in patients without *MYCN* amplification [10]. Tumors that present exclusively whole-chromosome copy number variations are associated with excellent survival, but tumors with any type of segmental chromosome alterations exhibit a high risk of relapse[11–13], which increases with the number of alterations[14].

The anatomical location of the primary tumor, which can be assessed by imaging methods, was also described as a prognostic factor [15–28]. However, the primary site was always reported according to the anatomical compartment (e.g., neck, chest, abdomen or pelvis) although NBs may arise from distinct sympathetic structures within a single compartment. A sympathetic anatomical classification might be more relevant in terms of prognosis than a simple compartmental anatomical classification.

Imaging techniques (CT-scan, MRI) recommended at diagnosis for disease staging[5, 29] allow identifying the tumor origin precisely, its volume, shape and its local extension and might therefore represent a non-invasive method to get relevant prognostic information. To the best of our knowledge, no study specifically investigated the relationships between imaging patterns of NBs and survival or other prognostic factors, especially the genomic profile.

The present study therefore investigated relationships between the anatomical origin of NBs along the sympathetic system, their imaging pattern, their genomic profile and patient outcome. As a secondary objective, this study assessed the accuracy of imaging for the identification of the primary tumor site compared to surgical and pathological findings.

## Patients and methods

The Institut Curie Institutional Review Board approved this study. Written informed consent was obtained from parents or guardians for inclusion in the clinical trials. Analysis of tumor samples was performed according to the relevant national law on the protection of participants in biomedical research. This retrospective observational study was conducted according to STROBE guidelines.

### Inclusion criteria

The following inclusion criteria were used: (1) patient age 18 years or younger at diagnosis, (2) patient referred to our institution before 2013, (3) cytologically or histologically proven NB, (4) frozen tumor material obtained at the time of diagnosis that enabled DNA extraction and molecular analysis, (5) availability of DICOM (Digital Imaging and Communications in Medicine) imaging data (i.e., CT scan or MRI) at diagnosis and during follow-up, (6) operative report availability in cases of surgery, and (7) availability of pathological reports of material obtained.

Our institutional database identified 203 eligible patients. DICOM data were missing for 56 patients, aCGH data for 11 patients and follow-up data for 3 patients. As a result, 133 patients were included for analysis.

### Clinical, surgical and pathological data

Clinical, surgical and pathological data was extracted from medical charts including surgical and pathological reports. INPC classification (i.e., “favorable” or “unfavorable” subtypes) derived from the pathological description at diagnosis, except when cellular material was obtained using only fine-needle aspirates.

All patients were treated using previous or ongoing protocols or trials of the International Society of Paediatric Oncology European Neuroblastoma (SIOPEN) (NCT 01704716, 00025428, 00025597, 00025649, 00025623, 00025610; details available in S1 File): 18 patients with localized disease had surgical removal of their tumor only, 86 patients were operated after neoadjuvant chemotherapy, and 27 patients were treated with chemotherapy without further surgery (either no local treatment or radiation therapy in case of high-risk tumors, i.e. INSS-stage 4 or INSS-stage 2–4 MNA tumors). Two patients had only clinical and radiological follow-up without any treatment.

### Imaging data analyses

**Fig 1:**
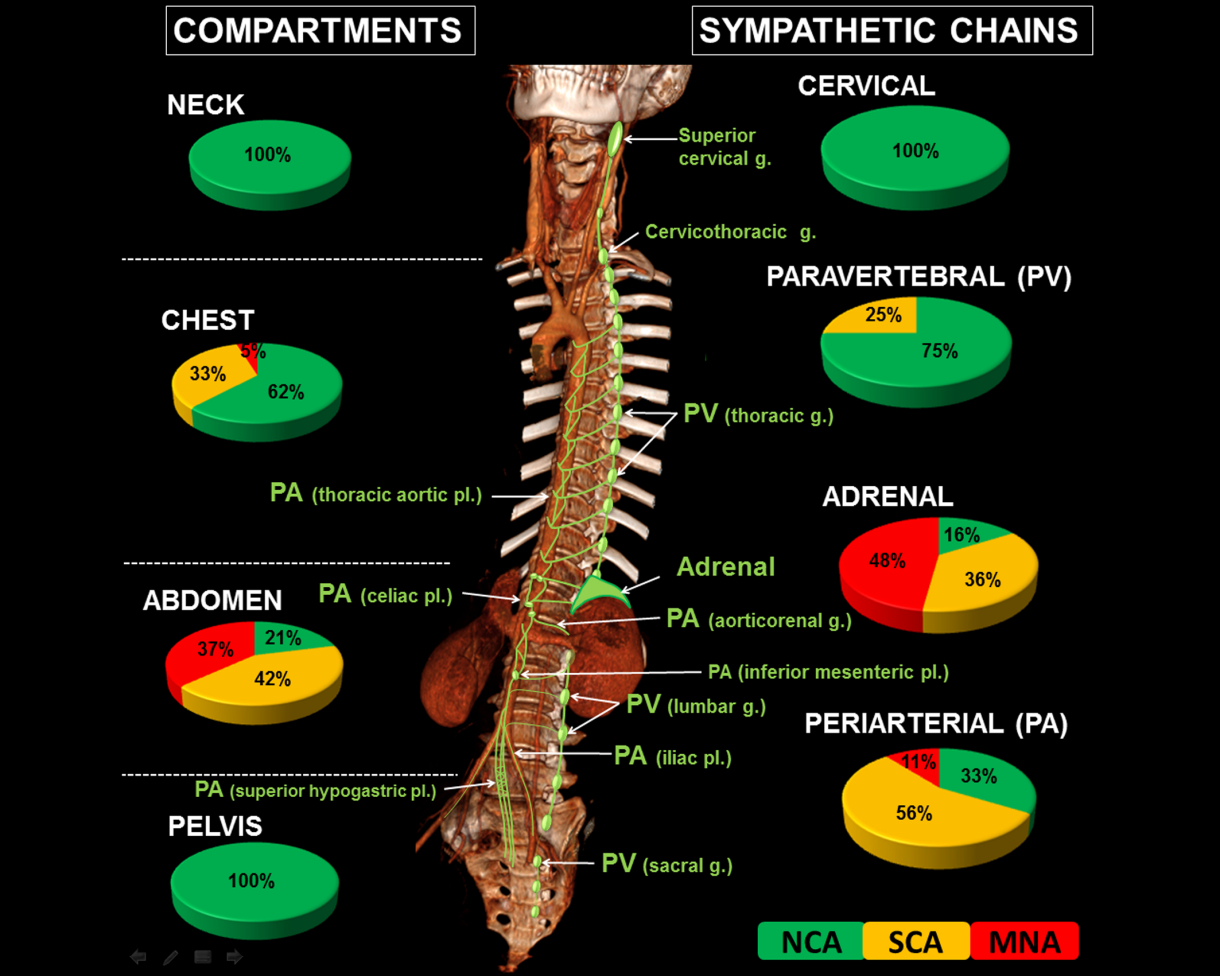
Radiogenomics classification of neuroblastomas according to anatomical origin. Neuroblastomas may be classified based on the anatomical compartment (i.e., neck, chest, abdomen or pelvis) or according to the sympathetic structure the tumors arise from, i.e., (1) the cervical sympathetic chains (i.e., including the superior, middle and inferior cervical and the cervicothoracic ganglia (g.)); (2) the paravertebral (PV) sympathetic chains (i.e., including all thoracic, lumbar and sacral ganglia); (3) the periarterial (PA) sympathetic pathways (i.e., including the thoracic aortic, abdominal aortic and celiac plexus (pl.)), the aorticorenal ganglia, and the superior and inferior mesenteric, superior hypogastric and iliac plexus); and (4) the adrenal glands. For each compartment or sympathetic group, the pie charts show the distribution of the genomic profile of the tumors, i.e., numerical-only chromosome alterations (NCA), segmental chromosome alterations (SCA) or *MYCN* amplification (MNA).

Imaging data was extracted from the Picture Archiving and Communicating System (PACS) of our institution (V 11.3, Carestream Health, Vaughan, Canada) and centrally reviewed in consensus by one senior pediatric radiologist (HJB, 20 years’ experience), two senior (SS, PPC, 20 and 15 years’ experience) and one fellow (TB, 3 years’ experience) pediatric surgeons who were blinded to any other data. Data included 207 CT scans and 62 MRI (24 patients had both examinations). Due to the retrospective nature of the study, images used for assessing tumor location and size were either enhanced-CT images, T1-weighted images (WI), T2-WI or contrast-enhanced T1-WI. Each tumor was classified as originating from one of the four anatomical compartments (i.e., neck, chest, abdomen or pelvis) and from one of the following sympathetic structure groups (according to the international Terminologia Anatomica): (1) cervical sympathetic chains (i.e., superior, middle and inferior cervical and cervicothoracic ganglia), (2) paravertebral sympathetic chains (i.e., thoracic, lumbar and sacral ganglia), (3) periarterial sympathetic pathways (i.e., thoracic aortic, abdominal aortic and celiac plexus, aorticorenal ganglia, superior and inferior mesenteric, superior hypogastric and iliac plexus), and (4) adrenal glands (Fig 1). NBs originating from lumbar ganglia and iliac plexus were classified as originating from the abdomen, and tumors originating from the superior hypogastric plexus (i.e., in the division angle of the aorta at L5 level) were classified in the pelvis. Tumor origin according to sympathetic anatomy was first assessed on imaging at diagnosis only and then compared to imaging after chemo-reduction and surgical and pathological data. The primary site was finally allocated by consensus on the basis of all available data.

Tumor volume at diagnosis was calculated from measurements in three perpendicular dimensions based on an elliptical estimate (volume = length x width x thickness x ᴨ/6). When preoperative chemo-reduction was used, preoperative tumor volumes and tumor volume decrease were calculated (Tumor volume decrease (%) = 100 x [1—Residual Volume / Initial Volume]). Tumor shape was classified as “single mass” or “multiple confluent masses”. Image-defined surgical risk factors (IDRFs) were assessed according to the published list and definition[29].

### Genetic analyses

The following genetic analyses were included: (1) the *MYCN* status assessed using fluorescence in situ hybridization (FISH) and an *MYCN* probe (Zymed Laboratories, San Francisco, CA, USA) on frozen sections for tumor fragments or cytogenetic preparations of fine-needle aspirates according to recommendations of the INRG Biology Committee[30], and (2) aCGH performed on BAC/PAC or NimbleGen arrays, as previously described[11, 14]. DNA was extracted from tumor specimens that were obtained at diagnosis and exhibited a tumor cellularity higher than 50%. The results were analyzed on the VAMP site using the GLAD algorithm and submitted to visual inspection[31, 32]. Three genomic types were defined: “numerical-only chromosomal alterations” (NCA) type, which only included numerical changes in whole chromosomes without any detectable structural rearrangement; “segmental chromosomal alterations” (SCA) type, which was characterized by any partial chromosome imbalances, excluding *MYCN* amplification, with or without associated numerical aberrations; and “*MYCN*-amplified” type (MNA), which exhibited *MYCN* amplification, with or without segmental or numerical aberrations[11].

### Statistical analysis

Comparisons between genomic profiles and continuous variables were performed using the non-parametric Kruskal–Wallis test. Comparisons between genomic profiles and discrete variables were performed using the Chi^2^ test or Fisher’s exact test, if necessary. Event-free survival (EFS) was defined as the time from diagnosis to first event (local or metastatic failure, or death). Patients with no events were censored at the time of last follow-up. Overall survival (OS) was defined as the time from diagnosis to death from any cause or last follow-up. Survival curves were analyzed using the Kaplan-Meier method and results were compared using the log-rank test. The 5-year rates were expressed together with their standard error (SE). For each variable, relative risks were estimated using a univariate Cox model and expressed with their 95% confidence interval. Relationships between anatomical origin and imaging pattern of neuroblastomas and survival were assessed by a multivariate analysis using a Cox model with a forward procedure. Multivariate analysis was performed among variables demonstrating significance by univariate analysis. Hence, the relationship between survival and the two anatomical classifications (compartmental and sympathetic) and tumor volume was assessed on OS only. P-values less than .05 were considered statistically significant. The anonymized data set necessary to replicate our study findings are at the European Genome-phenome Archive (study accession number: EGAS00001002651).

## Results

### Study cohort characteristics and relationships between patient age, INPC, stage and genomic profile

A total of 133 patients (73 males, 60 females) treated between 1998 and 2012 were included. Median age at diagnosis was 15 months (range, 0–151) (Table 1). Fifty-nine percent of the children were younger than 18 months at diagnosis. Fifty-one percent of the children had metastatic disease (63 children stage 4, 5 children stage 4S). Univariate analyses demonstrated that patient age, INPC and stage significantly correlated with the genomic profile, i.e., a NCA profile being observed more frequently in younger children, in NBs with favorable INPC and in lower stage disease (Table 1).

**Table 1:** Study cohort characteristics and relationships between patient age, INPC, stage and genomic profile.

Genomic profile^(1)^		NCA	SCA	MNA	Total	*P-value*
N =		*50*	*47*	*36*	*133*	
**Age at diagnosis** (months)	**≤ 18 m**	41	26	12	**79**	*< .0001*
	**> 18 m**	9	21	24	**54**	
	**Median age** (range)	6 (0–151)	16 (0–81)	25 (8–148)		*.0023*
**INPC**^(2)^	***Favorable***	*24*	*13*	*1*	38	*< .0001*
	***Unfavorable***	6	17	13	36	
**INSS Stage**	**1**	11	3	0	14	*< .0001*
	**2**	11	6	1	18	
	**3**	18	9	6	33	
	**4**	8	26	29	63	
	**4S**	2	3	0	5	
**INRG SS Stage**	**L1**	5	2	0	7	*< .0001*
	**L2**	35	16	7	58	
	**M**	8	26	29	63	
	**MS**	2	3	0	5	

(1) NCA: numerical-only chromosome alterations; SCA: segmental chromosome alterations; MNA: *MYCN*-amplification.

(2) Available data for only 74 of the 133 patients.

### Relationships between tumor origin, genomic profile and outcome

Both anatomical classifications, i.e., the compartmental one and the sympathetic one, significantly correlated with the genomic profile (Table 2, Fig 1).

**Table 2:** Relationship between anatomical primary tumor location (compartment and sympathetic origin) of neuroblastomas and their genomic profile.

Genomic profile^(1)^		NCA	SCA	MNA	Total	*P-value*
*N =*		*50*	*47*	*36*	*133*	
**Anatomic compartment**	**Neck**	9	0	0	**9**	*< .0001*
	**Chest**	13	7	1	**21**	
	**Abdomen**	20	40	35	**95**	
	**Pelvis**	8	0	0	**8**	
**Sympathetic origin**	**Cervical**	**9**	**0**	**0**	**9**	*< .0001*
	*upper cervical*	*4*	*0*	*0*	*4*	
	*stellate ganglion*	*5*	*0*	*0*	*5*	
	**Paravertebral**	**21**	**7**	**0**	**28**	
	*upper chest*	*8*	*5*	*0*	*13*	
	*lower chest*	*5*	*1*	*0*	*6*	
	*lumbar*	*3*	*1*	*0*	*4*	
	*presacral*	*5*	*0*	*0*	*5*	
	**Periarterial**	**9**	**15**	**3**	**27**	
	*mediastinal*	*0*	*1*	*1*	*2*	
	*celiac*	*0*	*4*	*0*	*4*	
	*pre-renal*	*4*	*6*	*1*	*11*	
	*median subrenal*	*1*	*2*	*1*	*4*	
	*Zuckerkandl organ*	*0*	*1*	*0*	*1*	
	*sup*.*hypog*. *plexus*	*3*	*0*	*0*	*3*	
	*iliac*	*1*	*1*	*0*	*2*	
	**Adrenal gland**	**11**	**25**	**33**	**69**	

(1) NCA: numerical-only chromosome alterations; SCA: segmental chromosome alterations; MNA: *MYCN* amplification.

According to the compartmental anatomical classification, all tumors arising from the neck or pelvis were NCA NBs. In the chest, 62% were NCA, 33% SCA and only 5% MNA. Abdominal tumors were more widely distributed with 21% NCA, 42% SCA and 37% MNA. Eighty-five percent of SCA and 97% of MNA NBs were observed in the abdomen.

With regards to their sympathetic structure origin, all tumors arising from the cervical sympathetic chains were NCA. Among paravertebral tumors, 75% were NCA, 25% SCA and none MNA. Among periarterial tumors, 56% were SCA, 33% NCA and 11% were MNA. The distribution of genomic profiles was wider among adrenal tumors: 16% were NCA, 36% SCA and 48% MNA. Ninety-two percent of MNA NBs originated from the adrenal gland.

**Fig 2:**
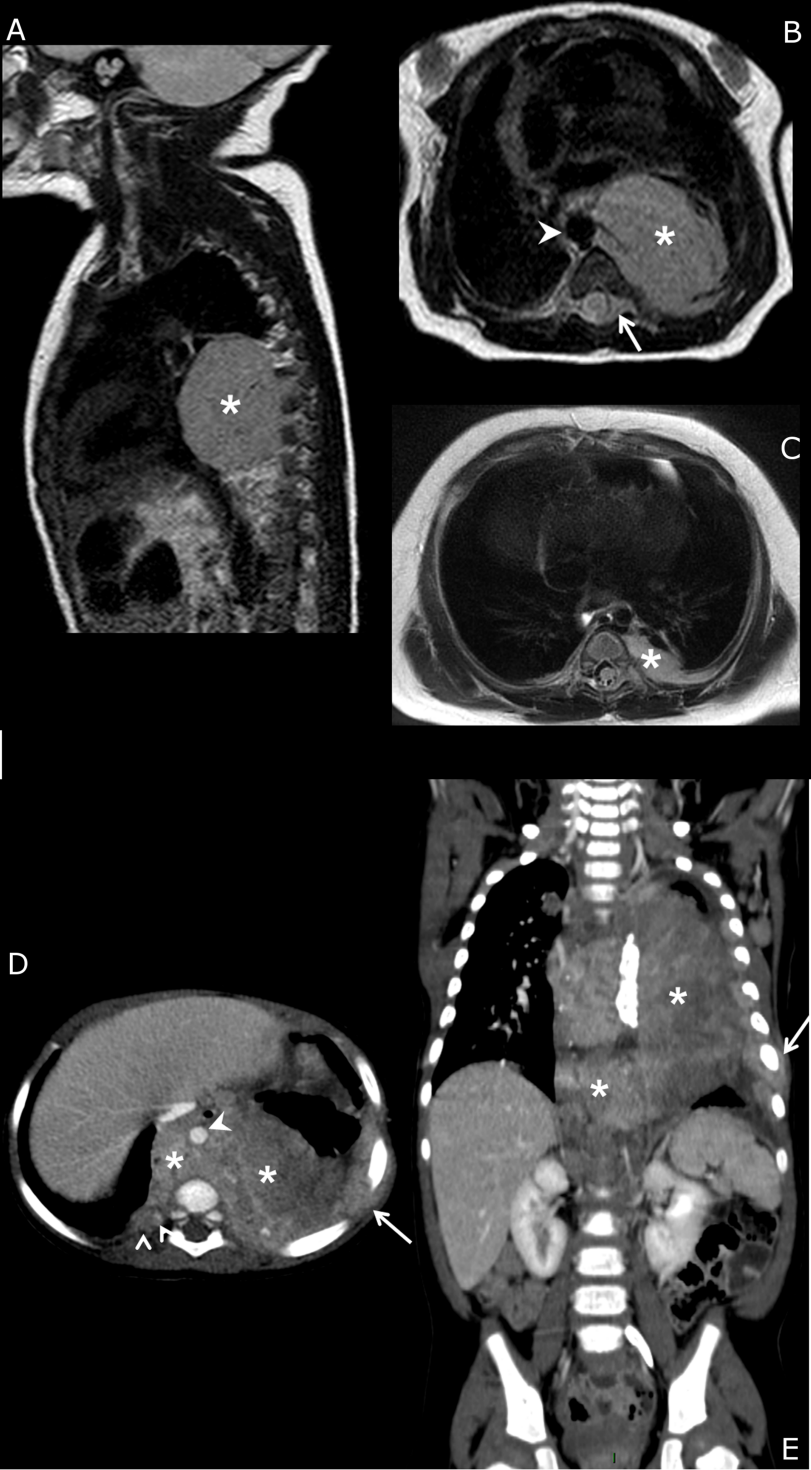
Imaging phenotypes of chest neuroblastomas. **(a, b, c)** Newborn with L2-stage left posterior **mediastinal NCA neuroblastoma**. MRI at diagnosis (a, b: sagittal and axial T2-weighted sequences). The primary tumor is a unique well-delineated mass (*) with focal contact with the thoracic aorta (arrowhead) and intra-spinal extension (arrow). Follow-up MRI 3 months later (c) after neoadjuvant chemotherapy (2 courses of cyclophosphamide-vincristine and 2 courses of etoposide-carboplatin) shows the tumor residue precisely located at the costo-vertebral junction, i.e., a paravertebral sympathetic chain location. **(d, e)** 9-year-old girl with M-stage **mediastinal SCA neuroblastoma**. Enhanced CT scan at diagnosis (axial and coronal views). The primary tumor (*) is ill-defined and diffusely infiltrates the posterior mediastinum, pleura and chest wall (arrows), crosses the midline and encases the thoracic aorta (arrowhead). The presumed origins are the mediastinal sympathetic fibers surrounding the descending aorta.

**Fig 3:**
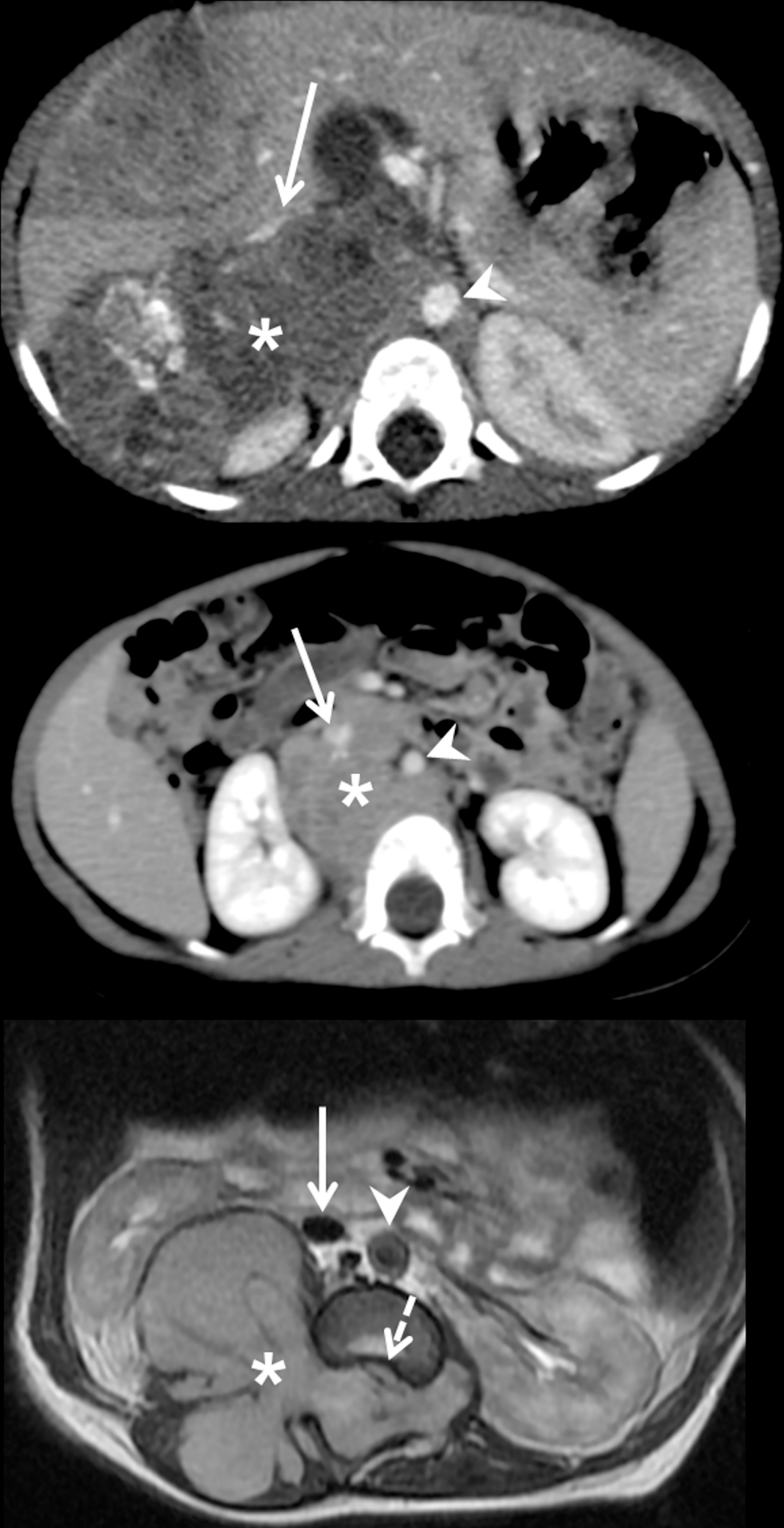
Imaging phenotypes of abdominal neuroblastomas. **(a)** 18-month-old girl with M-stage right **adrenal MNA neuroblastoma**. Enhanced CT scan at diagnosis. The primary tumor (*) is centered on the right adrenal gland between the right kidney and the inferior vena cava (arrow) and extends medially in contact with the aorta (arrowhead). **(b)** 12-month-old girl with M-stage retroperitoneal **periarterial SCA neuroblastoma**. Enhanced CT scan at diagnosis. The primary tumor (*) is centered in the median retroperitoneum around the aorta (arrowhead) and behind the inferior vena cava (arrow). **(c)** Newborn with L2-stage lumbar dumbbell **paravertebral NCA neuroblastoma**. Axial T2-weighted MRI at diagnosis. The primary tumor (*) is centered on the right paravertebral chain and invades the psoas and spinal muscles and fills the spinal canal, compressing the spinal cord (dotted arrow). The tumor is totally separated from the inferior vena cava (arrow) and the aorta (arrowhead).

Within a single anatomic compartment, the sympathetic chain or structure from which NBs originated allowed differentiation of tumors with distinct biological features. Ninety percent (19/21) of chest tumors arose from the paravertebral chains (Table 2, Fig 2A and 2B) and these tumors were mostly NCA (68%) or SCA (32%), whereas the two tumors that originated from the mediastinal periarterial sympathetic pathways were SCA and MNA, respectively (Fig 2D and 2E). Within the abdomen, 73% (69/95) of NBs were of adrenal origin (Fig 3A), 23% (22/95) were periarterial (27% NCA, 64% SCA and 9% NMA) (Fig 3B) and 4% (4/95) were lumbar paravertebral (3 NCA and 1 SCA) (Fig 3C). Among pelvic tumors, 62.5% (5/8) were presacral paravertebral NBs and 37.5% (3/8) periarterial (superior hypogastric plexus) and all were NCA NBs.

**Fig 4:**
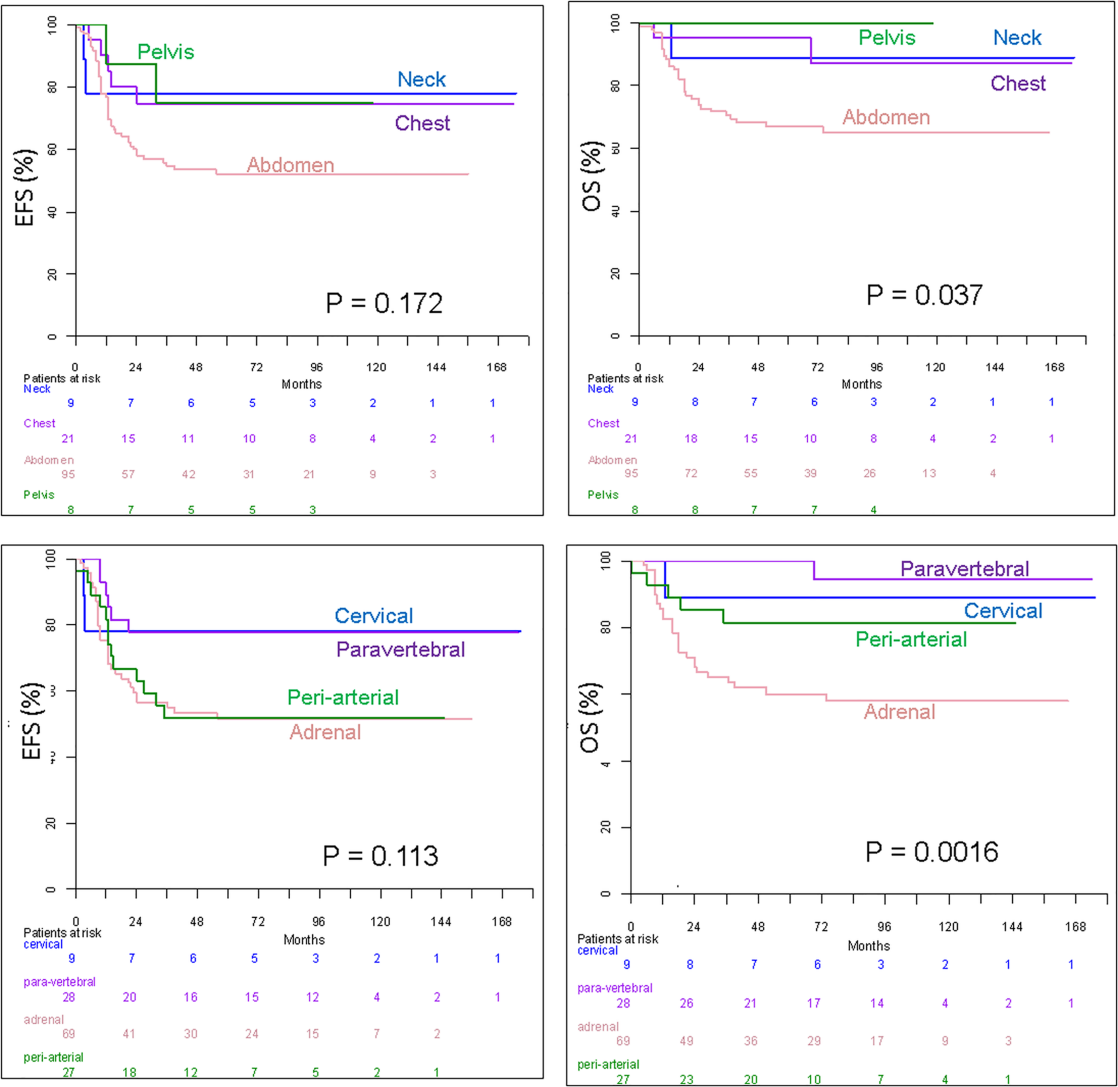
Kaplan-Meier survival analysis according to anatomical classifications of primary tumors. Event-free survival (EFS) and overall survival (OS) according to the anatomical compartment (a, b) and sympathetic system origin (c, d) of the primary tumor.

The anatomical origin of NBs also significantly related to outcome (Table 3, Fig 4). The compartmental classification revealed that abdominal NBs exhibited a significantly lower 5-year OS and a trend of lower 5-year EFS than extra-abdominal primaries. Survival was not significantly different between neck, chest and pelvic tumors. The sympathetic classification demonstrated that adrenal NBs had a significantly lower 5-year OS (and a trend of lower 5-year EFS) compared to extra-adrenal primary tumors. EFS was not significantly different among the four sympathetic origin groups (P = .113). No significant EFS difference was observed between cervical and paravertebral NBs (P > .87) on one hand, neither between adrenal and periarterial NBs (P > .90) on the other hand. However, pooled NBs originating from adrenal gland *or* periarterial sympathetic chains had significantly lower EFS that pooled NBs originating from cervical *or* paravertebral chains (P < .0015). Multivariate analysis demonstrated that the sympathetic anatomic classification was significantly more relevant than the compartmental one for the prediction of OS (P < .0003).

**Table 3:** Relationships between imaging phenotypes of neuroblastomas and outcome (univariate analysis).

		N	5Y-EFS^(1)^ ± SE	RR^(2)^_95%_CI	*P-value*	5Y-OS^(1)^ ± SE	RR^(3)^_95%_CI	*P-value*
**Anatomical compartment**	Neck	9	77.8 ± 13.9	2.22 [1.08–4.53]^(4)^	.*17*	88.9 ± 10.5	4.79 [1.47–15.64] ^(4)^	.*037*
	Chest	21	74.7 ± 9.8			95 ± 4.9		
	Abdomen	95	52.1 ± 5.2			66.9 ± 4.9		
	Pelvis	8	75 ± 15.3			100		
**Sympathetic system origin**	Cervical	9	77.8 ± 13.9	1	.*11*	88.9 ± 10.5	1	.*0016*
	Paravertebral	28	77.6 ± 8.1	0.90 [0.18–4.44]		100	0.31 [0.02–5.02]	
	Periarterial	27	51.9 ± 9.6	2.19 [0.49–9.72]		81.5 ± 7.5	1.75 [0.2–15.01]	
	Adrenal	69	51.4 ± 6.1	2.29 [0.55–9.57]		60.2 ± 6	4.29 [0.58–31.52]	
**IDRF**	No	11	72.7 ± 13.4	1	.*37*	90.9 ± 8.7	1	.*21*
	Yes	122	57.3 ± 4.6	1.69 [0.53–5.41]		73.1 ± 4.1	3.35 [0.46–24.48]	
**Tumor Volume**^(5)^	≤ 160 mL	66	64 ± 6	1	.*23*	83.8 ± 4.7	1	.*0088*
	> 160 mL	66	53.8 ± 6.2	1.39 [0.8–2.39]		64.9 ± 5.9	2.52 [1.23–5.14]	
**Tumor volume decrease**	≤ 50%	16	50 ± 12.5	1 [0.44–2.28]	.*58*	87.5 ± 8.3	0.39 [0.00–1.32]	.*11*
	50–89%	53	59.6 ± 6.9	0.74 [0.4–1.37]		76.7 ± 5.9	0.53 [0.26–1.09]	
	≥ 90%	42	52.3 ± 7.7	1		59.3 ± 7.6	1	

(1) EFS: event-free survival; OS: overall survival; SE: standard error. The mean follow-up of the cohort was 83 months (range, 1–175 mo). The 5-year EFS and OS of the entire cohort were 58.6% (+/- 4.3%) and 74.6% (+/- 3.8%), respectively.

(2) RR: relative risk of events; CI: confidence interval.

(3) RR: relative risk of death.

(4) Relative risk has been estimated in abdominal tumours compared to cervical, thoracic or pelvic tumours, as no death occurred in patients with pelvic tumours (RR could not be estimated by the Cox model for this subgroup of patients). For homogeneity of results, the same cluster has been done for estimating EFS’ hazard ratio. However, Logrank tests compare survival and EFS of the 4 anatomical compartments.

(5) (1 missing data).

### Contribution of initial imaging to the classification of tumor origin according to sympathetic system anatomy

Initial imaging was judged relevant to allocate the sympathetic origin of the primary in 77% (102/133) of cases. Review of imaging after chemotherapy (Fig 2C) modified the interpretation of tumor origin in 14% of cases. Surgical and pathological reports provided additional relevant anatomical details that were not depicted by imaging at diagnosis in 11% and 12% of cases, respectively. The mean tumor volume of patients for whom post-chemotherapy imaging, surgical or pathological reports provided additional information was significantly higher than that of patients with anatomical location that was correctly judged on initial imaging (362 mL versus 223 mL, P = .0087). The anatomical compartment in the former group was mainly the abdomen (25/31), and the sympathetic origin was primarily the adrenal gland (14/31) or periarterial pathways (10/31).

### Relationships between tumor imaging pattern (IDRFs, shape, volume, tumor volume decrease), genomic profiles and outcome

The occurrence of any IDRF was not significantly related to the genomic profile. However, the median number of IDRFs among the 122 IDRF-positive tumors was significantly higher in MNA NBs (Table 4). The 5-year OS and EFS rates of IDRF-negative patients were higher than IDRF-positive patients but these differences were not significant (Table 3).

**Table 4:** Relationships between IDRFs, tumor shape, initial volume, tumor volume decrease and genomic profile.

Genomic profile^(1)^		NCA	SCA	MNA	Total	*P-value*
	*N =*	*50*	*47*	*36*	*133*	
**IDRF**	0	7	3	1	11	.*19*
	+	43	44	35	122	
**IDRF number**^(2)^ (median, range)		2 (1–8)	3 (1–13)	5 (1–14)		*< .0001*
**Tumor shape**	single mass	42	26	16	84	.*0003*
	multiple confluent masses	8	21	20	49	
**Tumor volume** at diagnosis median (range) in mL		69 (3–553)	151 (1–1040)	524 (14–1626)		*< .0001*
**Tumor volume decrease** median (range)		79% (0%, 100%)	81% (39%, 99%)	96% (38%, 99%)		*< .0001*
Number of patients treated with neoadjuvant chemotherapy		37	41	35		

(1) NCA: numerical-only chromosome alterations; SCA: segmental chromosome alterations; MNA: *MYCN*-amplification.

(2) IDRF number among the 122 IDRF-positive NBs.

The “single mass” pattern was significantly more frequent than the “multiple confluent masses” pattern in NCA (84%) compared to SCA and MNA NBs (55% and 44%, respectively).

Mean tumor largest diameter and volume at diagnosis were 8.5 cm and 255 mL, respectively. A tumor volume of 160 mL (median volume) or less at diagnosis was significantly associated with better OS, but not significantly better EFS. Initial tumor volume of MNA NBs was significantly higher than NCA and SCA NBs. The tumor volume decrease after neoadjuvant chemotherapy was significantly higher in MNA tumors but was not significantly associated with survival.

## Discussion

NBs in a single anatomical compartment may derive from distinct sympathetic structures which are associated with distinct genomic profiles. Therefore, the precise anatomical origin of the primary tumor is of special interest. We described statistically significant relationships between the sympathetic origin of tumors and their genomic profile and outcome. Our data also confirmed that initial imaging efficiently depicted the sympathetic origin of the tumor compared to surgical and pathological findings, except for very large abdominal masses for which post-chemotherapy imaging was more accurate, i.e., when the tumor shrinks on its original sympathetic structure.

It has long been suggested that tumor behavior may differ based on the primary tumor location and adrenal NBs are known to be associated with poorer prognosis[33]. Other studies enhanced this concept by comparing the primary tumor site with patient survival rates or other prognostic factors, such as tumor stage, histology and serum markers[17, 27]. Chest NBs are associated with better outcome among the extra-abdominal sites[16, 23]. However, multivariate analysis in a large retrospective study[20] did not identify the chest location as an independent prognostic factor. Cervical and pelvic NBs are also associated with better prognosis, although these results were based on smaller series[15, 18, 19, 22, 24, 25]. The INRG recently confirmed that adrenal tumors were more likely than non-adrenal tumors to have MNA, and thoracic tumors were less likely than non-thoracic tumors to have MNA[28]. Our whole-genome DNA copy number analysis allowed the identification of significant relationships between cervical and pelvic locations and NCA profile, and between chest location and non-MNA NBs. It also confirmed the strong association between abdominal location and MNA profile and poorer outcome.

By using a sympathetic anatomical classification for the first time instead of the classical compartmental one, our study provided a better differentiation of outcome. This result is explained by the occurrence of distinct genomic profiles within each compartment. The recognition of a paravertebral sympathetic origin is notable because these tumors are not associated with MNA type and mostly associated to the favorable NCA profile (75% in this series). Imaging identifies paravertebral tumors as arising in the chest (“costo-vertebral” NB), abdomen (“lumbar” tumors) or pelvis (“presacral” tumors), possibly associated with intra-spinal extension (“dumbbell” tumors). Imaging also identifies periarterial tumors, which are observed in various compartments, primarily the abdomen around the aorta or its branches, and occasionally in the pelvis (superior hypogastric plexus). In the chest, the use of the sympathetic classification allows differentiation between periarterial mediastinal and paravertebral tumors. Although more widely distributed, genomic profiles of periarterial NBs were less favorable than those of paravertebral NBs, including 56% SCA and 11% MNA types. Finally, the adrenal gland was the origin of most MNA NBs (92% in this series).

Together our data supports the hypothesis that genomic profiles and the aggressiveness of NBs may be associated with distinct neural crest cell-derived elements. During embryogenesis, neural crest cells emerge early in development and a defined region gives rise to precursor cells that differentiate into the adrenal medulla and sympathetic ganglia[2]. The exact mechanisms that lead to tumorigenesis are not fully determined. According to our data, it is remarkable that the most distally (i.e., cervical and presacral) and dorsally (i.e., paravertebral) migrating cells are mostly associated with the favorable NCA genomic profile, whereas adrenal and periarterial tumors are more associated with the less favorable SCA and MNA profiles.

Our study also demonstrated links between tumor volume, shape, tumor volume decrease and genomic profile and outcome. A small tumor volume was significantly associated with non-MNA NBs and better survival. A single mass was more frequently associated with favorable NCA NBs than multiple confluent masses. The link between IDRFs and outcome remains controversial in the literature[34, 35]. We did not identify a significant link with genomic profile or outcome among localized tumors, but this result may be related to insufficient statistical power. However, a high number of IDRFs was significantly related to the MNA type. Tumor volume reduction during the early phase of induction chemotherapy in high-risk NBs was reported as associated with a better outcome[36]. The tumor volume decrease in this study, which included any risk-group NBs, was not associated with survival, and MNA NBs were associated with a higher tumor volume decrease compared to SCA and NCA tumors.

We acknowledge that our study includes limitations. As the primary sympathetic structure is usually distorted by the tumor, the exact origin of the tumor may be difficult to assess. The reference anatomical location used in this study was based on imaging data obtained before and after neoadjuvant chemotherapy, as well as on the macroscopic and microscopic descriptions from the surgeons and pathologists. For adrenal tumors, the residual gland invaded by tumor cells and surrounding the tumor was helpful in defining the tumor origin. For other locations, we were only able to define the most probable sympathetic chain involved. Other limitations of our work are the relatively small size of our cohort and the non-inclusion of highly differentiated NBs, such as intermixed ganglioneuroblastomas and ganglioneuromas, for which aCGH profiles are not contributive. Actually, the quality of aCGH interpretation is directly correlated to tumor cells content and the assessment of the exact balance between immature neuroblastic cells, ganglion cells, Schwann cells and stroma is challenging in mature tumors. Very few samples reach the minimum threshold of tumor cells content required (i.e., 50%). Actually, normal cells and subclonal populations tend to lessen the dynamic of genomic profiles which can be totally flat. When aCGH fits all quality controls but shows a flat profile, the result is tagged as “not contributive”. However, prognostic information is less useful for those tumors that share a comparable good prognosis[37]. Functional imaging was not addressed because of the retrospective nature of the study. Early results using diffusion-weighted MRI suggested that neuroblastoma and ganglioneuroma / ganglioneuroblastoma might be differentiated using this method[38]. Among metastatic NBs, 123-I-MIBG scan was recently used to differentiate MNA from non-MNA NBs[39]. Functional imaging may actually provide additional diagnostic and prognostic information in the future.

## Conclusion

Imaging phenotypes of neuroblastomas correlate with tumor genomic profile and patient outcome. If confirmed in a larger study cohort, the combination of anatomical data (sympathetic structure origin) and morphological pattern (volume, shape, number of IDRFs) may represent relevant prognostic criteria. In addition, our data potentially suggest distinct tumor genesis pathways according to the neural crest cells origin that could contribute to a better understanding of the observed genomic profiles.

## Supporting information

S1 File Supporting information on treatment regimens. (DOCX)
