# Supplementary material for: Radiogenomics of neuroblastomas: Relationships between imaging phenotypes, tumor genomic profile and survival
Source: PLoS One. 2017 Sep 25;12(9):e0185190. doi: 10.1371/journal.pone.0185190 (PMC5612658; doi:10.1371/journal.pone.0185190)
Supplement: S1 File — (DOCX) [file pone.0185190.s001.docx]

**Supplemental Patient Information: Information on treatment protocols**

Patients treated in centers of the Société Française des Cancers de l’Enfant (SFCE) in France were treated according to the following national or international treatment protocols: for infants less than 1 year at diagnosis until 2014 INES99 ^1-3^ (Clinical Trial No: NCT 00025597, 00025649, 00025623, 00025610) and from 2014 LINES (NCT 01728155, <https://clinicaltrials.gov/ct2/show/NCT01728155>); for patients with localized resectable disease, LNESG2 ^4^; for patients > 1 year of age with localized unresectable disease until 2014, EUNB ^5^ (NCT 00025428) and from 2014 LINES (NCT 01728155); for patients with metastatic disease, HR-NBL-01^6^ (NCT 01704716).

All clinical trials received approval from an ethic committee.

These treatment protocols have used a uniform risk stratification at diagnosis, taking into account age at diagnosis, INSS stage, tumor resectability in localized disease, as well as *MYCN* amplification. In brief, in case of a localized, resectable tumour, patients were treated by surgery only. In infants with localized unresectable disease, neoadjuvant chemotherapy consisted of Vincristine-Cyclophosphamide for patients without life threatening symptoms, and of Etoposide-Carboplatine in case of life threatening symptoms. Infants with stage 4s disease were either observed only (in the absence of life threatening symptoms), or received chemotherapy as above (in the presence of life threatening symptoms). Infants with stage 4 disease received chemotherapy using Etoposide-Carboplatine and Vincristine-Cyclophosphamide-Adriamycine. Children older than 1 year with localized unresectable disease received neoadjuvant chemotherapy based on Etoposide-Carboplatine and Vincristine-Cyclophosphamide-Adriamycine, surgery, as well as radiotherapy in some cases. Finally, children older than 1 year at diagnosis with stage 4 disease or with tumors harboring *MYCN* amplification received induction chemotherapy using the same drugs as above, then high dose chemotherapy based on alkylating agents followed by autologous stem cell reinjection, surgery, local radiotherapy and instances maintenance therapy using retinoic acid, and more recently also immunotherapy.

**References :**

1. Rubie, H. et al. Excellent outcome with reduced treatment in infants with nonmetastatic and unresectable neuroblastoma without MYCN amplification: results of the prospective INES 99.1. *J Clin Oncol* **29**, 449-55 (2011).

2. De Bernardi, B. et al. Excellent outcome with reduced treatment for infants with disseminated neuroblastoma without MYCN gene amplification. *J Clin Oncol* **27**, 1034-40 (2009).

3. Canete, A. et al. Poor survival for infants with MYCN-amplified metastatic neuroblastoma despite intensified treatment: the International Society of Paediatric Oncology European Neuroblastoma Experience. *J Clin Oncol* **27**, 1014-9 (2009).

4. Cecchetto, G. et al. Surgical risk factors in primary surgery for localized neuroblastoma: the LNESG1 study of the European International Society of Pediatric Oncology Neuroblastoma Group. *J Clin Oncol* **23**, 8483-9 (2005).

5. Kohler, J.A. et al. Treatment of children over the age of one year with unresectable localised neuroblastoma without MYCN amplification: results of the SIOPEN study. *Eur J Cancer* **49**, 3671-9 (2013).

6. Ladenstein, R. et al. Randomized Trial of prophylactic granulocyte colony-stimulating factor during rapid COJEC induction in pediatric patients with high-risk neuroblastoma: the European HR-NBL1/SIOPEN study. *J Clin Oncol* **28**, 3516-24 (2010).
